# Supplementary material for: Conformational dynamics of the ABC transporter McjD seen by single‐molecule FRET
Source: EMBO J. 2018 Sep 20;37(21):e100056. doi: 10.15252/embj.2018100056 (PMC6213277; doi:10.15252/embj.2018100056)
Supplement: Supplementary file 1 — Expanded View Figures PDF [file EMBJ-37-e100056-s001.pdf]

Expanded View Figures

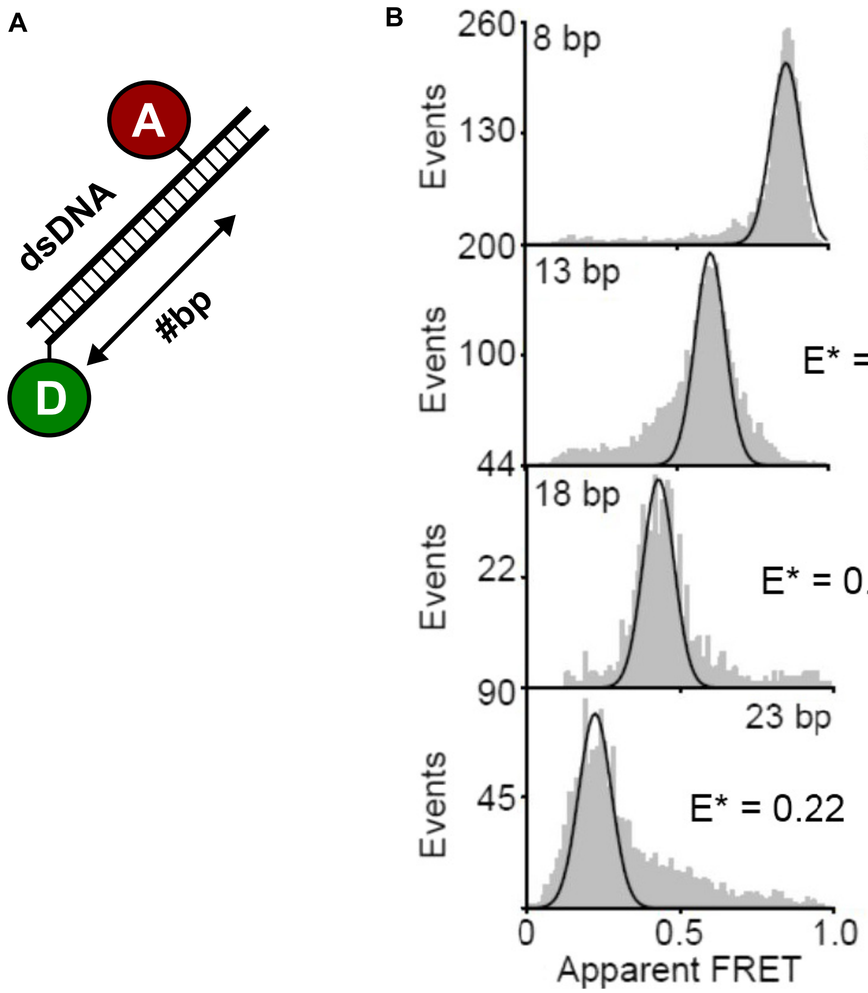

Figure EV1. Static dsDNA control experiments.

A Schematic representation of the smFRET assay using labeled double-stranded DNA with Alexa 555 and Cy5 fluorophores as indicated. Distance estimates can be derived by multiplication of 0.34 nm times the number of base pairs; these provide only an inaccurate approximation of the distance between donor and acceptor dye.

B Experimental data similar to those shown in Fig 2 are shown, which support the validity of experimental results and interpretations given in the main text.

 © 2018 The Authors

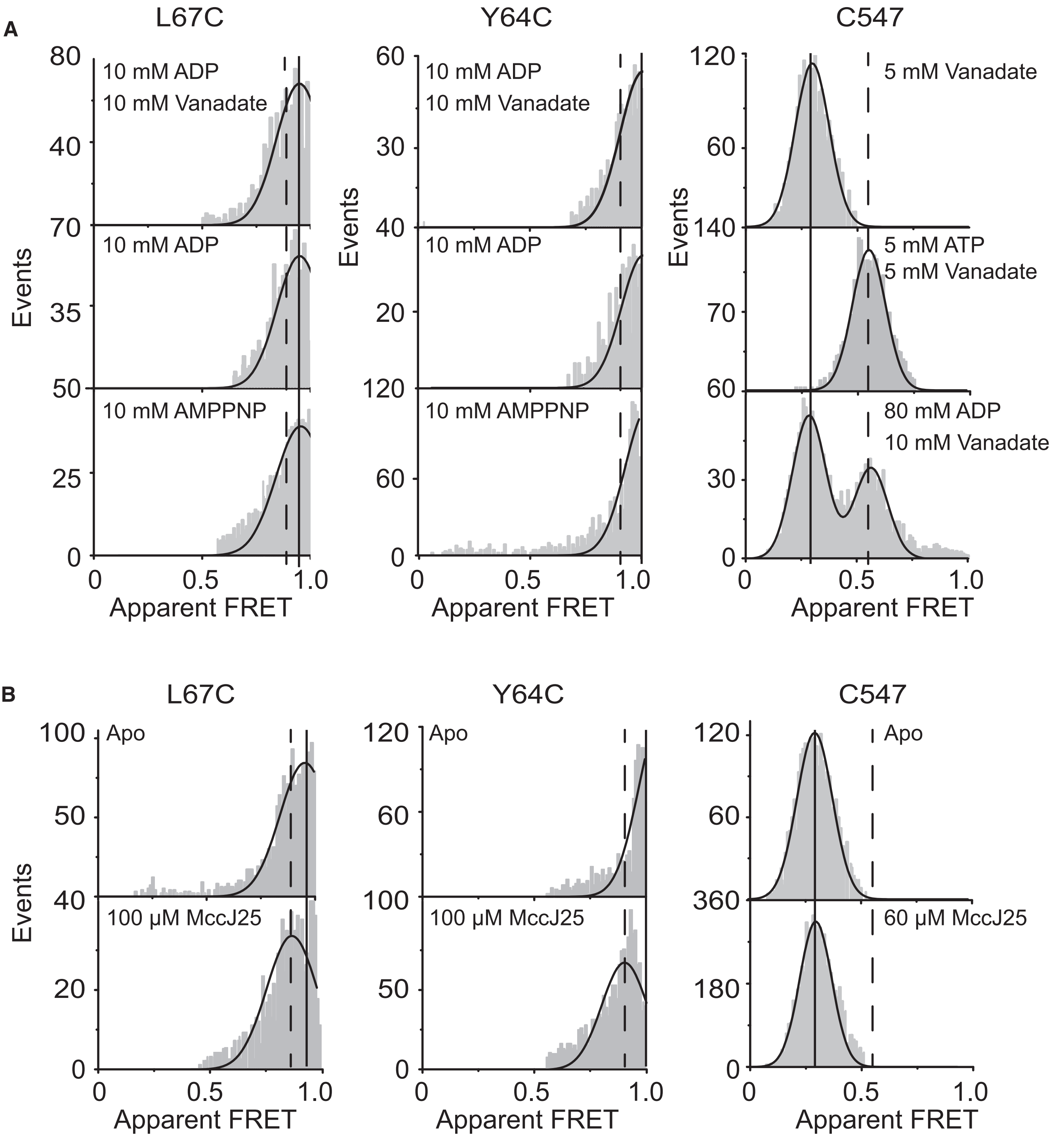

Figure EV2. Additional data for detergent-solubilized McjD.

A, B Confocal single-molecule analysis of labeled McjD and variants in detergent using ALEX. Mean values of ligand-free conditions are shown as a reference with a solid marker, while ligand-bound mean values (A) are shown dashed (compare Fig 2). (B) Mean values of ligand-free conditions are shown as a reference with a solid marker, while ligand-bound mean values are shown dashed. Note that the two panels cannot be compared directly due to slightly different microscope alignment.

© 2018 The Authors

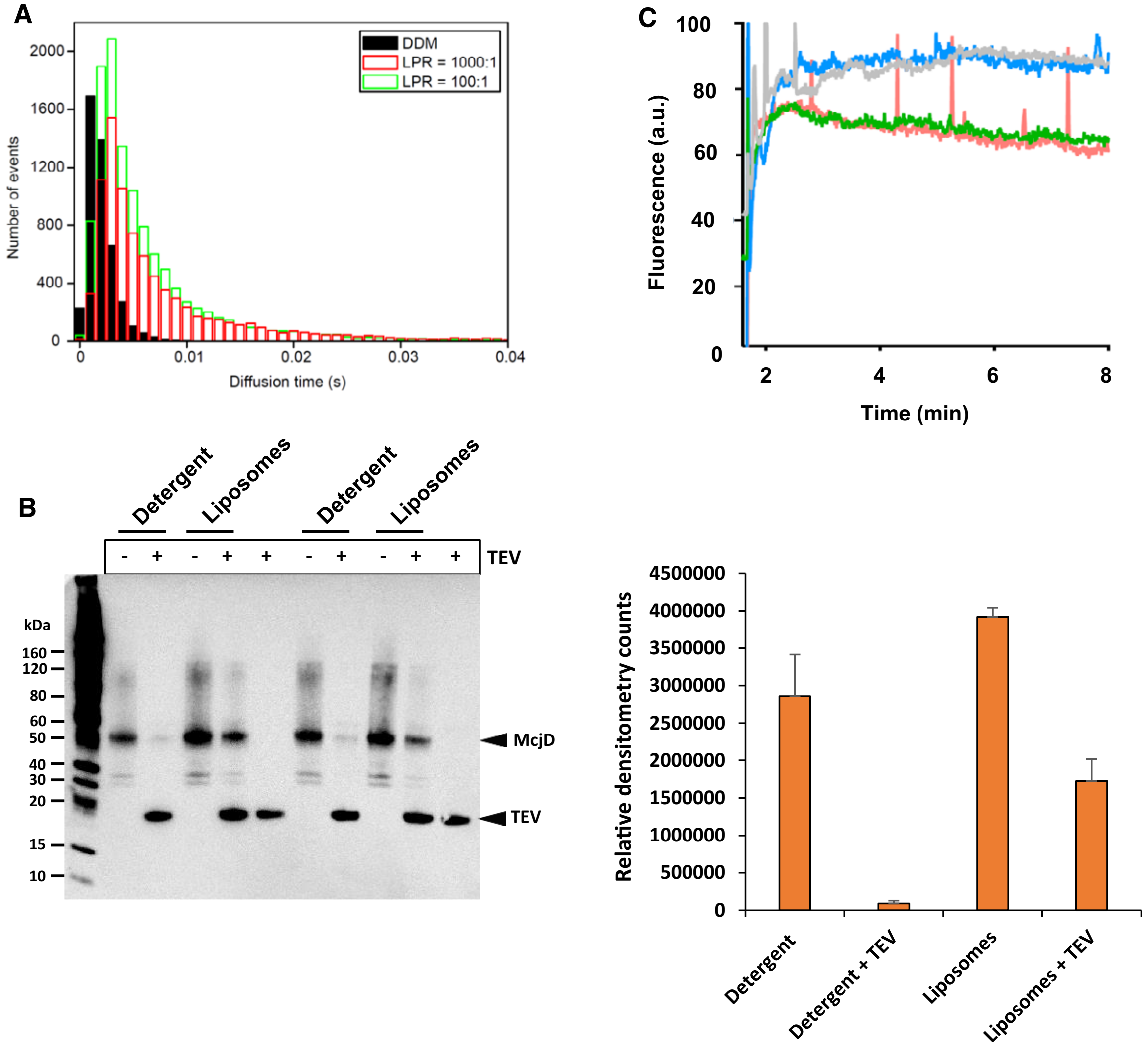

Figure EV3. Support for liposome incorporation of McjD into proteoliposomes.

A Comparison of mean burst length for detergent-solubilized McjD and reconstitution conditions. The mean burst length is directly related to the diffusion time of the protein complex. While the data show successful incorporation into liposomes, it additionally indicates that different protein-to-lipid ratios did not have a significant effect on the reconstitution efficiency.

B Western blot showing the orientation of McjD in proteoliposomes (left panel). Detergent-purified McjD-His (~50 kDa) and liposome-reconstituted McjD-His were incubated with TEV protease (~20KDa), which carries a His-tag, at a 1:1 molar ratio of protein to TEV. The reactions were visualized before and after TEV cleavage by anti-His Western blot. The McjD-His-tag is located at the C-terminus of the NBDs. McjD-His molecules facing the interior of the liposomes are inaccessible for TEV cleavage, whereas the ones facing outside are accessible for cleavage. Comparison of the TEV-treated and untreated McjD-His-containing liposomes shows that cleavage is not complete, suggesting that McjD-His has inserted in the liposomes in different orientations. As a control experiment, detergent-purified McjD-His (~50 KDa) can no longer be detected after TEV treatment suggesting full removal of the His-tag. The reaction conditions for each lane are shown above the gel. Bands corresponding to McjD-His and TEV protease-His are labeled. Densitometry quantification of McjD orientation (right panel). The ratio of inside to outside McjD-His in liposomes was quantified by measuring the Western blot band intensities before and after TEV protease incubation. Values represent mean densitometry counts calculated from two independent liposome preparations and indicate a proportion of 44%:56% ± 4% McjD-His inside to outside. Error bars are shown (mean ± standard deviation, n = 2).

C McjD mediates the ATP-dependent uptake of Hoechst in proteoliposomes (green line); no uptake is observed in the absence of ATP (grey line). The labeled C547 mutant displayes similar Hoechst transport activity (red line with ATP and blue line without ATP) as the wild type McjD.

© 2018 The Authors

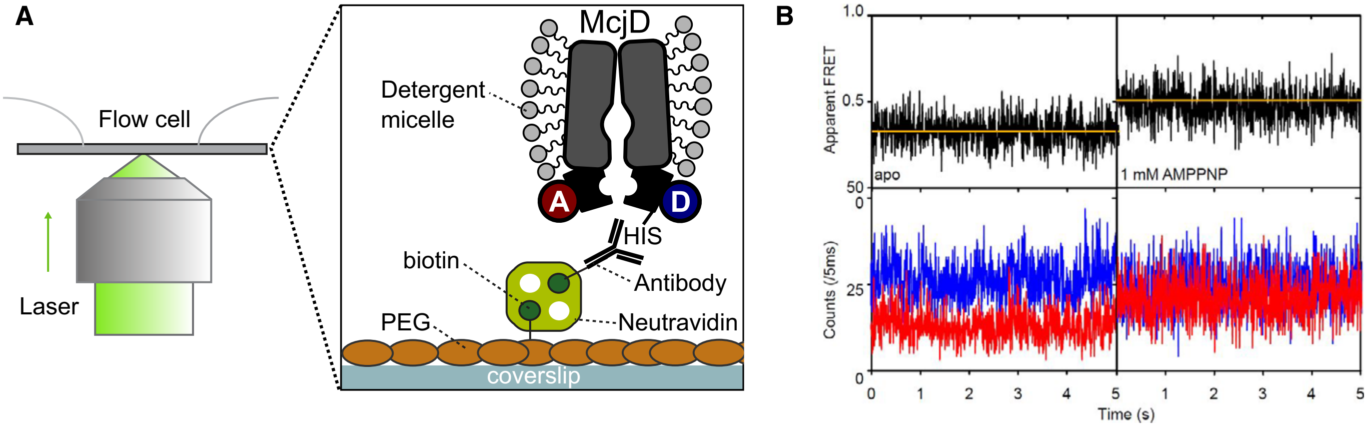

Figure EV4. Conformational states of surface-immobilized C547-McjD in detergent environment.

A Schematic cartoon of the immobilization of C547-McjD to the surface via PEG-biotin and an antihistidine antibody.

B Data from confocal scanning experiments show representative fluorescence time traces (blue, donor signal; red, acceptor signal; black, FRET signal; yellow, fit) of McjDs labeled with Alexa555 and Alexa647-maleimide at indicated concentrations of corresponding substrate AMPPNP and apo conditions.

© 2018 The Authors
